# Impact of respiratory muscle training on clinical and functional parameters in COVID-19 recovered patients

**DOI:** 10.11604/pamj.2023.46.65.36615

**Published:** 2023-10-19

**Authors:** Imen Sahnoun, Tasnim Znegui, Ines Moussa, Siwar Rejeb, Yosor Ayedi, Sirine Hattab, Salma Mokaddem, Saloua Jameleddine, Leila Douik El Gharbi

**Affiliations:** 1 University of Tunis El Manar, Tunis Medical School, Pneumology Department D, Abderrahmen Mami Hospital, Ariana, Tunisia,; 2 University of Tunis El Manar, Tunis Medical School, Epidemiology and Statistics Department, Abderrahmen Mami Hospital, Ariana, Tunisia,; 3 University of Tunis El Manar, Tunis Medical School, Department of Physiology, Abderrahmen Mami Hospital, Ariana, Tunisia

**Keywords:** Post-COVID-19, respiratory rehabilitation, persistent symptoms

## Abstract

**Introduction:**

early respiratory rehabilitation is required for patients with coronavirus virus disease 2019 (COVID-19) sequelae to reduce the risk of serious disabilities after hospital discharge.

**Methods:**

it was a comparative prospective study including patients with persistent symptoms one month after discharge. The patients were hospitalized at the pneumology department D of Abderahman Mami hospital for COVID-19 pneumonia. The study involved two groups: (G1) included patients who participated in respiratory muscle training program (twice a week during 6 weeks), and a control group (G2). The groups were matched based on age, sex and body mass index (BMI). Persistent symptoms and pulmonary lung function (forced vital capacity (FVC), forced expiratory volume in one second (FEV1), total lung capacity (TLC) and diffusion capacity for carbon monoxide (DLCO), maximal inspiratory pressure (PI max) and maximal expiratory pressure (PE max), 6 Minute Walk distance (6-MWD) at baseline and after 6 weeks were compared between the two groups.

**Results:**

the two groups of patients were comparable in terms of age, sex, BMI, comorbidities, and extent of lung computed tomography (CT) lesions. Compared to G2, a significant improvement of persistent symptoms was noted in G1, including dry cough (p=0.002), dyspnea (p=0.001), chest pain (p=0.002), and fatigue (p=0.001). The mean of percutaneous oxygen saturation (SpO2) increased from 96.68% to 97.93% (p<0.01) in G1. A significant improvement in the percentages of change of FEV1 (p=0.005), FVC (p=0.003), TLC (p<0.001), DLCO (p<0.001), and 6-MWD (p=0.015) was also noted in G1 after this program. Nevertheless, only the percentage of FEV1 (p=0.02) increased in the control group. No impact of respiratory muscle training on PI max and PE max was noted.

**Conclusion:**

the present study demonstrated a significant improvement of persistent symptoms and exercise tolerance after short-term respiratory muscle training in patients suffering from COVID-19 sequelae.

## Introduction

The global pandemic caused by SARS-CoV-2 has affected millions of people. After the acute phase of coronavirus disease 2019 (COVID-19), survivors have faced the risk of prolonged COVID-19, defined as the development or persistence of symptoms once the acute phase of the disease is over [1]. Long-term sequelae, including fatigue, breathlessness and lung impairment, such as restrictive ventilation disorders and compromised diffusion capability, are largely reported by several studies [2]. The long-term effects in COVID-19 survivors are of great concern and they may have a significant impact on their quality of life. The pathological mechanisms underlying the disease remain unknown.

However, persistent inflammation is considered a key mediator in the multifactorial genesis of the long-term sequelae [3,4]. It has been demonstrated that pulmonary rehabilitation, defined as a comprehensive non-pharmacological intervention, is highly effective in managing dyspnea and increasing exercise capacity in chronic respiratory diseases, such as chronic obstructive pulmonary diseases and interstitial lung diseases [5]. Given the burden of this disease, policy statements and position papers have suggested acute and long-term rehabilitation as a strategy to improve patient outcomes [6,7]. Rehabilitation can therefore play a crucial role in restoring function and limiting disability. A prospective study was conducted to evaluate the clinical and functional impact of respiratory muscle training in patients with persistent respiratory impairment after COVID-19.

## Methods

**Study design:** it was an evaluative prospective study, conducted from June 2020 to February 2021, including patients hospitalized at the pneumology department D of Abderahman Mami hospital (Tunisia) for COVID-19 pneumonia. All patients had a follow-up appointment at the hospital one month after discharge. Written informed consent for participation was obtained from all the patients included in this study after receiving a comprehensive description of the program. The study was approved by the local Ethics Committee of Abderahman Mami hospital (Tunisia) (N: 07/2021).

**Applied definition:** post-acute sequelae of COVID-19 defined as symptoms persisting beyond the 4-week period of acute infection [8].

**Inclusion criteria:** patients aged 18 years or above, hospitalized for moderate or severe SARS-Cov2 pneumonia and suffering from post-acute sequelae of COVID-19 after discharge. Forced expiratory volume in 1 s (FEV1) ≥ 70%.

**Exclusion criteria:** patients with moderate or severe heart disease, ischemic or hemorrhagic stroke or neurodegenerative diseases, locomotor impairment, and chronic respiratory failure. Refusal to participate.

**Study procedure:** epidemiological data (age, sex, body mass index (BMI), smoking, and comorbidities), clinical data of acute COVID-19 infection and extent of lung Computed Tomography (CT) lesions, were collected from the patients' medical records. Persistent symptoms were obtained using a standardized questionnaire.

For evaluation, a questionnaire was used to reveal persistent symptoms, including dyspnea, cough, fatigue, and chest pain. The modified Medical Research Council dyspnea scale (mMRC) and the Borg Dyspnea Scale were used to assess dyspnea. The visual analog scale (VAS) was used to assess chest pain. Percutaneous oxygen saturation (SpO2) and respiratory rate were measured.

Body plethysmography was performed. The following parameters related to respiratory function were measured: forced vital capacity (FVC), (FEV1), FEV1/FVC ratio, total lung capacity (TLC) and diffusion capacity for carbon monoxide (DLCO), inspiratory muscle strength (PI max, a measure of inspiratory muscle strength), and expiratory muscle strength (PE max, a measure of abdominal and intercostal muscle strength). Exercise capacity was measured using 6-min walking test (6-WMT).

The study involved 2 groups: An intervention group (G1) included patients who participated in respiratory muscle training program and a control group (G2). The groups were matched based on age, sex and BMI. For the control group (G2), persistent symptoms and respiratory function at baseline and 6 weeks after inclusion were compared. For the intervention group (G1), persistent symptoms and respiratory function at baseline and 6 weeks after applying the program of respiratory muscle rehabilitation were compared.

The program of respiratory rehabilitation based on chest physiotherapy involving respiratory muscle training, cough exercise, diaphragmatic training, stretching exercises, breathing exercises, and home exercises was applied [9]. The program was conducted twice a week over a six-week period under the supervision or assistance of a specialized physiotherapist. In the process of respiratory rehabilitation, patients should act according to their abilities. Physiotherapists need to closely monitor the patients' vital signs. The patients' oxygen saturation should be greater than 93% and their heart rate should be less than 120 beats/minute during the session.

**Statistical analyses:** statistical analyses were performed using SPSS software version 26. Qualitative variables were described by percentages. Normality was verified by Shapiro wilk test. If distribution was normal, quantitative variables were described by means and standard deviation (SD); otherwise, they were described by medians and Interquartile range (IQR). Comparison of percentages between the groups was conducted using Chi Square test. Comparison of means between T0 and T6 was performed using Wilcoxon test. Results were obtained by applying repeated measures analysis of variance (Friedman or Kruskal-Wallis ANOVA). Significant threshold was set at 0.05 for all analyses.

## Results

A total of 42 patients were included (20 males, 22 females). G1 included 30 patients and G2 12 patients. No differences were noted between the two groups with regard to age, sex, BMI, comorbidities, and extent of lung (CT) lesions (Table 1). The persistent symptoms noted in the present study were: dyspnea (n=29, 69.05%), chest pain (n=17, 40.48%), fatigue (n=17, 40.48%), and dry cough (n=15, 35.71%).

**Table 1:** baseline characteristics of COVID-19 patients

Characteristic	All patients	G1	G2	p
	(n=42)	(n=30)	(n=12)	
**Age (years), mean (SD)**	59.38 (±14)	60.23 (±14)	57.25 (±13)	0.55
**Sex, n (%)**				
Men	20 (47.6)	14 (46.7)	6 (50)	0.84
Women	22 (52.4)	16 (53.3)	6 (50)	
**Smokers, n (%)**	1 (2.5)	1 (3.3)	0 (0)	1
**BMI (kg/m2), mean (SD)**	30.1 (5.98)	29.46 (5.6)	31.85 (6.8)	0.26
**Comorbidity, n (%)**				
Hypertension	20 (48.8)	13 (43.3)	7 (58.3)	0.24
Obesity	20 (48.8)	13 (43.3)	7 (63.6)	0.24
Diabetes mellitus	8 (19.5)	6 (20)	2 (18.2)	0.63
Asthma	2 (4.9)	2 (6.7)	0 (0)	1
Coronary heart disease	2 (4.9)	1 (3.3)	1 (9.1)	0.47
**Duration of symptoms prior to admission (day), median (IQR)**	9 (7-13)	9.5 (6-13)	9 (8-14)	0.925
**Extent of lung CT lesions >50%,n (%)**	14 (34.1%)	9 (30%)	5 (41.7%)	0.35
**Length of hospitalization, day, median (IQR)**	10 (8-15.75)	10 (8-16)	11 (7.5-15.5)	0.43

SD: standard deviation, BMI: Body Mass Index, IQR: Interquartile range, CT: computed tomography

The baseline functional parameters were as follows: FEV1 (89.35%), FVC (85.73%), TLC (77.70%), FEV1/FVC (79.53%), DLCO (65.67%), PI max (-66.7), PE max (78.78) and 6-min walking distance (6 MWD) (485.68m±119). A significant improvement in dyspnea, cough, fatigue, and chest pain was noted in G1 after 6 weeks (Table 2). The mean SpO2 increased from 96.68% to 97.93% (p<0.01) in G1. The mean respiratory rate decreased from 21.64 to 19.39 in G1 (p= 0.02) and from 25.63 to 20.88 (p=0.01) in G2 (Table 3).

**Table 2:** comparison of persistent symptoms in both groups at baseline and after 6 weeks

	G1 (n=30)		p	G2 (n=12)		p
	Pre	Post		Baseline	After 6 weeks	
**Dry cough n (%)**	13 (43.8)	1 (3.4)	0.002	2 (22.2)	1 (11.1)	1
**Dyspnea, n (%)**	22 (75.9)	4 (13.8)	<0.001	7 (77.8)	5 (55.6)	0.50
**mMRC, median (IQR)**	2 (1-3)	0	<0.001	2 (1-2)	1 (0-2)	0.18
**Borg, median (IQR)**	2.5 (2-5)	0 (0-1)	<0.001	1.5 (0-2)	0 (0-1)	0.157
**Chest pain, n (%)**	13 (44.8)	3 (10.3)	0.002	4 (44.4)	3 (33.3)	1
**Fatigue, n (%)**	14 (48.3)	1 (3.4)	0.001	3 (33.3)	2 (22.2)	1

mMRC: Modified Medical Research Council Dyspnea Scale; IQR: Interquartile range.

**Table 3:** comparison of lung function and exercise capacity in both groups at baseline and after 6 weeks

	G1 (n=30)		p	G2 (n=12)		p	Factorial ANOVA	
	Pre	post		Baseline	After 6 weeks		F	p
**FEV1(L)**	2.49	2.64	<0,001	2.56	2.78	0,042	0,061	0,806
**FEV1 (% pred)**	93.7	99.10	0,003	81.43	87.43	0,017	0,11	0,741
**FVC(L)**	3.04	3.2	0,001	3.06	3.14	0,397	0,104	0,748
**FVC (% pred)**	89.6	95.86	0,002	79.43	82.71	0,088	0,036	0,851
**FEV1/ FVC (%)**	82.67	81.47	0,139	81.83	85.35	0,116	1,963	0,166
**TLC (L)**	4.34	4.67	<0,001	3.96	4.54	0,043	0,016	0,899
**TLC (% pred)**	80.33	85.56	0,002	76.57	82	0,352	0,276	0,599
**DLCO (% pred)**	66	74	<0.001	76	80	0,397	0,07	0,792
**6-MWD (m), mean (SD)**	466±139	517±90	<0,001	526±50	504±90	0,018	0,584	0,477
**FR (cycles/min)**	21.64	19.39	0,013	25.63	20.88	0,018	0,298	0,587
**SpO2 (%)**	96.68	97.93	0,001	97.63	97.38	0,48	2,906	0,093
**PI max (cmH2O)**	-75	-74.5	0,181	-57.29	-104.71	0,176	2,093	0,153
**PE max (cmH2O)**	81	85	0,107	80.29	87.57	0,866	0,001	0,97

FEV1: forced expiratory volume in one second, FVC: forced vital capacity, TLC: total lung volume, DLCO: diffusion capacity, PI max: maximal inspiratory pressure, PE max: maximal expiratory pressure, SpO2: percutaneous oxygen saturation FR: respiratory rate, 6 -MWD: 6-minute walk distance.

Participants in G1 showed a significant improvement in FEV1 (L, %), FVC (L, %), TLC (L, %), DLCO (%), and 6-MWD after this program. However, only FEV1 (%) increased significantly in the control group. No significant improvement was noted in both G1 and G2 with regard to respiratory muscles strength (Table 3). No severe adverse events were noted.

## Discussion

The persistent symptoms revealed by the present study included dyspnea (69.05%), chest pain (40.48%), fatigue (40.48%), and dry cough (35.71%). Indeed, 10-20% of the patients reported at least one persistent symptom one month after acute COVID-19 infection [10]. Many appellations have been used to describe persistent symptoms once the acute phase of COVID-19 is over, such as post-acute sequelae of COVID-19, ongoing symptomatic COVID-19, post-acute COVID-19 syndrome, and prolonged COVID-19 [1,11]. A list of over 200 different symptoms in the evolution of COVID-19 sequelae has been reported [1,12]. A systematic review and meta-analysis reported that the most prevalent respiratory symptoms in survivors after COVID-19 infection are fatigue, dyspnea, chest pain, and cough with 52%, 37%, 16%, and 14%, respectively [13].

In the present study, a decrease in both TLC and DLCO with significant change was noted. Indeed, the most common lung function abnormalities reported by studies are diffusing capacity impairment followed by restrictive ventilatory impairment. These sequelae are correlated to the severity of the acute disease [14,15]. The pathological mechanisms underlying the disease and its impact on clinical symptoms are still largely unknown. Host genetic factors, organ damage, and viral mechanisms may be involved in COVID-19 sequelae [16]. In a prospective, longitudinal study, evaluating COVID-19 survivors by magnetic resonance imaging, researchers reported the possibility of residual organ impairment, especially the heart and lungs, as the cause of COVID-19 sequelae [17]. These sequelae can lead to decreased exercise tolerance, thus affecting the development of normal activity [12].

Management of patients with prolonged COVID-19 remains a challenge. Some expert consensus and guidelines, published by the World Health Organization [18], Chinese Medical Association of Rehabilitation [19], and European Respiratory Society/American Thoracic Society [20], recommend early bedside in-hospital rehabilitation and regular daily activities after hospital discharge for patients with COVID-19. However, no specific recommendations have been made regarding the optimal program of pulmonary rehabilitation [20]. Rehabilitation program after discharge is deemed necessary to improve persistent symptoms, lung function, and the quality of life [21]. This study compared the control group, significant clinical improvement in fatigue, dyspnea, cough, and chest pain following short-term respiratory muscle training which was noted in G1. An increase in oxygen saturation was also noted in G1. These results were similar to those reported by Sun *et al*. [22] demonstrating an increase in the activities of daily living score and a relief in patients' cough and dyspnea after only 3 weeks of rehabilitation. Similar findings were reported in the study of Rayegani *et al*. [23]. In their study involving 36 elderly patients with COVID-19 who participated in a 6-week respiratory training consisting of strength training, cough exercise, and diaphragmatic training, Liu *et al*. reported an improvement in anxiety, depression, and quality of life scale [24].

Several studies have demonstrated that pulmonary rehabilitation improves SpO2 in survivors with COVID-19 [7,9,22,25]. In this study, 6-week respiratory muscle rehabilitation significantly improved all functional parameters, including FEV1, FVC, TLC, DLCO, and 6 MWT in COVID-19 survivors. Nevertheless, only the percentage of FEV1 increased significantly in G2. In a prospective observational cohort study including 50 patients after the acute phase of moderate and critical COVID-19 infection, Liu *et al*. [24] demonstrated a significant improvement in respiratory function. Moreover, Gloeckl *et al*. [25] showed an improvement in 6-MWD, FEV1, and FVC with no correlation with the disease severity. Indeed, recruitment of respiratory intercostal muscles and abdominal wall muscles has important contributions to preserving respiratory function [24].

A systematic review and meta-analysis, including 3 studies published on February 2022, showed a beneficial effect of rehabilitation on 6-WMD in 233 participants. The between-group difference in change of 6-WMD was significant in the experimental group (50.41m, 95%; p < 0.0001). However, a systematic review published in April 2022, demonstrated inconsistent results with regard to pulmonary function. However, significant improvements in muscle strength, walking capacity, sit-to-stand performance, and quality of life were reported [26,27]. Indeed, exercise attenuates immunosenescence is beneficial for immunological health. It is well-known that exercise is also an essential therapeutic tool for improving cardiovascular health and enhancing the vascular system [1,28].

In patients requiring long-term hospitalization, respiratory muscular atrophy and diaphragmatic dysfunction are very common [29]. Particularly, diaphragmatic dysfunction is detrimental. Its pathogenesis is complex and could be related to mitochondrial dysfunction [6]. The effects of this virus on the diaphragm are not completely explored. In a study conducted by Hayden *et al*. [30] the group with severe COVID-19 pneumonia showed a statistically significant improvement in PI max after 3 weeks of rehabilitation. However, in the present study, no significant improvement in PI max was noted, which may be due to the relatively short duration of the respiratory rehabilitation program or the requirement of an intensive diaphragmatic training. Therefore, individualized rehabilitation programs are required. They should be personalized based on comorbidities, age, obesity, and severity of sequelae [31]. To the best of the authors' knowledge, this study was the second Tunisian study evaluating the impact of respiratory muscle rehabilitation on patients with post-acute sequelae of COVID-19.

Our study had two limitations. First, it was a single-center study with a small sample size. Secondly, it involved only respiratory muscle training which is not considered as complete pulmonary rehabilitation, defined as a comprehensive intervention according to the American Thoracic Society and the European Respiratory Society [5]. To overcome these limitations, multicenter double-blind studies with complete rehabilitation programs are required.

## Conclusion

More attention has been paid to patients suffering from long-term COVID-19 sequelae. This study showed the benefits of respiratory muscle rehabilitation in terms of reducing fatigue and dyspnea, and improving lung function and exercise tolerance.

### What is known about this topic

- *Long-term sequelae, including fatigue, breathlessness, and lung impairment after the acute phase of COVID-19 are largely reported by studies;*
- *The assessment of post-COVID19 syndrome is considered an emerging challenge;*
- *Given the rapidly increasing burden of persistent symptoms, policy statements and position papers have proposed respiratory rehabilitation as a strategy to improve long-term outcomes of COVID-19 infection*.

### What this study adds

- *The present study demonstrated that pulmonary rehabilitation was a safe and effective therapeutic option in prolonged COVID-19;*
- *Rehabilitation of patients after COVID-19 recovery improves their functional status;*
- *It showed the benefits of respiratory muscle training in terms of reducing fatigue and dyspnea, and improving lung function and exercise tolerance in patients with persistent COVID-19 symptoms*.
